# Clinical features of pulmonary arterial hypertension associated with systemic sclerosis

**DOI:** 10.3389/fmed.2023.1264906

**Published:** 2023-09-27

**Authors:** Tijana Tuhy, Paul M. Hassoun

**Affiliations:** Division of Pulmonary and Critical Care Medicine, Department of Medicine, Johns Hopkins University School of Medicine, Baltimore, MD, United States

**Keywords:** pulmonary arterial hypertension, pulmonary hypertension, systemic sclerosis, scleroderma, pulmonary vascular disease

## Abstract

Systemic sclerosis is an autoimmune disorder of the connective tissue characterized by disordered inflammation and fibrosis leading to skin thickening and visceral organ complications. Pulmonary involvement, in the form of pulmonary arterial hypertension and/or interstitial lung disease, is the leading cause of morbidity and mortality among individuals with scleroderma. There are no disease-specific therapies for pulmonary involvement of scleroderma, and pulmonary arterial hypertension in this cohort has typically been associated with worse outcomes and less clinical response to modern therapy compared to other forms of Group I pulmonary hypertension in the classification from the World Symposium on Pulmonary Hypertension. Ongoing research aims to delineate how pathologic microvascular remodeling and fibrosis contribute to this poor response and offer a window into future therapeutic targets.

## 1. Overview of scleroderma

Systemic sclerosis (SSc) is a complex disorder characterized by abnormal collagen deposition, endothelial dysfunction, and immune dysregulation leading to tissue fibrosis (1–4). SSc classically has two distinct phenotypes, limited and diffuse cutaneous systemic sclerosis, distinguished by the degree of skin involvement. Visceral organ involvement can complicate either form of the disease. Further, overlap syndromes exist with other connective tissue diseases (5). The 2013 American College of Rheumatology/European League Against Rheumatism Classification Criteria for Systemic Sclerosis provide a sensitive scoring system for the clinical diagnosis of SSc, including the presence of skin thickening proximal to the metacarpophalangeal joints, fingertip lesions, telangiectasia, abnormal nailfold capillaries, pulmonary arterial hypertension (PAH) and/or interstitial lung disease, Raynaud’s phenomenon, and scleroderma-related auto-antibodies (including against RNA polymerase III, centromere, and topoisomerase I) (6).

Systemic sclerosis has a prevalence worldwide of approximately 17.6 in 100,000, with an annual incidence of 1.4 in 100,000 person-years (7). This belies significant geographical variation of the disease; among indigenous Canadian populations, the prevalence is as high as 47 in 100,000. Further, there is relatively low prevalence among East Asian populations as compared to European, North and South Americans (8). As with most autoimmune diseases, SSc affects women more frequently than men, with an estimated ratio ranging from 3:1 to 7:1 female: male predominance (9). However, men with SSc are more likely to have severe disease, more diffuse cutaneous manifestations, and greater degree of visceral organ involvement (8, 9).

Systemic sclerosis is an incurable disease whose diagnosis is associated with a reduced life expectancy; studies have reported median survival of 12 years (10). Renal disease was historically associated with the highest mortality, however, treatment and prevention of scleroderma renal crisis with angiotensin-converting enzyme inhibitors has led to the emergence of pulmonary disease as the major cause of morbidly and mortality (11).

## 2. Genetic basis for scleroderma

While the etiology is poorly understood, compelling data exist to support a genetic basis for SSc (12). A family history of disease is thought to represent the largest risk factor for development of SSc, with a 15-fold increase in relative risk for siblings (13, 14). Many SSc-associated gene-loci have been described, however many of these are shared with other autoimmune diseases. Candidate gene association studies have determined single nucleotide polymorphisms (SNPs) of functional significance in genes involved in adaptive immunity, innate immunity, and extracellular matrix regulation (15). A systemic review by Jiang et al. identified the following genetic and epigenetic factors specifically associated with PAH in SSc: *MIF* rs755622*C allele frequency, *TLR2* Pro631His variant, *UPAR* rs344781 gene variant, *KCNA5* single-nucleotide polymorphisms, lack of minor allele C and HLA-B35+ (16). Notably, while mutations in *BMPR2* are associated with most cases of familial PAH and some sporadic cases, they have not been found among two small cohorts of patients with SSc-PAH (17–19).

Genome-wide association studies have confirmed that the major histocompatibility complex (MHC) is the strongest susceptibility loci for SSc and described key culprit signaling pathways, chemokines and cytokines associated with SSc. A recently published meta-analysis of over 10,000 individuals with systemic sclerosis described 27 independent genome-wide associated signals, including molecular pathways linked to vasculopathy (*DDX6*, regulates VEGF under hypoxic conditions) and to fibrosis (*TWSG1*, involved in transforming growth factor-β signaling in T cells) (20). This work offers promising insights into pathogenesis and possible disease-modifying drug targets.

## 3. Pulmonary complications

**FIGURE 1:**
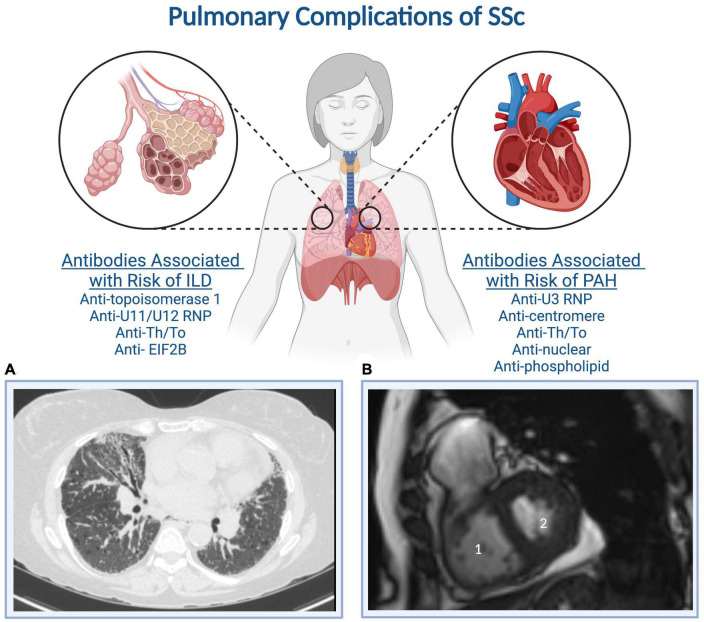
Systemic sclerosis auto-antibodies associated with the development of interstitial lung disease and pulmonary arterial hypertension. **(A)** A representative CT of the chest demonstrates subpleural cystic changes, interstitial thickening, and traction bronchiectasis, while **(B)** a representative cardiac MRI demonstrates (1) right ventricular dilation and (2) a D-shaped left ventricle indicative of RV pressure-volume overload. Created with BioRender.com.

Involvement of the pulmonary parenchyma and circulation frequently complicate SSc and can develop at any time during the disease course (12, 21, 22). Figure 1 shows auto-antibodies associated with pulmonary complications of SSc and representative imaging findings (23, 24). Pulmonary complications are the leading cause of death among individuals with SSc (10, 11). PAH, defined as a mean pulmonary artery pressure (mPAP) greater than 20 mm Hg and pulmonary vascular resistance (PVR) greater than 2 Wood units, is a progressive disease characterized by pathologic pulmonary vascular remodeling leading to right ventricular failure and death (25). SSc-associated PAH (SSc-PAH) is diagnosed by right heart catheterization (RHC) and occurs among 8–12% of individuals with SSc. It is one of the leading causes of pulmonary hypertension within group I PAH, and the leading cause of PAH within the group of connective tissue diseases in most Western world registries of PAH (while systemic lupus erythematosus is more common than SSc as the cause of PAH in China) (26, 27).

Patients with SSc-PAH are overwhelmingly female, mirroring trends in SSc. In men, SSc-PAH is usually diagnosed at a later age; these patients have more severe disease and worse outcomes. SSc-PAH carries a worse prognosis compared to other forms of Group 1 PAH. Patients with SSc-PAH have decreased survival compared to SSc patients without pulmonary hypertension and a worse response to therapy and lower survival compared to patients with idiopathic PAH (IPAH), with a median survival of less than 4 years in the pre-combination therapy era (28–30). Late age at diagnosis, the presence of pericardial effusion, worse functional severity by NYHA functional class, and hyponatremia also portend a worse prognosis (28, 31, 32). A more recent study shows promising improvement in survival, which is attributed, in part to earlier detection, but clearly combination therapy with two or more PAH specific drugs (33).

Beyond the pulmonary circulation, individuals with SSc often develop SSc-associated interstitial lung disease (SSc-ILD). Prominent SSc-ILD is frequently associated with topoisomerase 1 (Scl-70) antibody positivity (34). Diagnosis is made with pulmonary function testing (PFTs) and high-resolution computed tomography (HRCT). PFTs will demonstrate restrictive lung disease with low total lung capacity (TLC) and low single breath diffusing capacity to carbon monoxide (DLCO) (35). Characteristic ill-defined, subpleural infiltrates can be seen in the posterior lower lobes on HRCT, as can interstitial reticular infiltrates and subpleural honeycombing (35). Later disease may progress to traction bronchiectasis and cystic lung disease.

A third subset of patients develop both ILD and PH (SSc-PH-ILD), which is associated with the worst prognosis of the three forms (36, 37). SSc-PH-ILD is a form of WSPH group 3 pulmonary hypertension along with other forms of PH associated with parenchymal lung disease. Diagnosis is made with HRCT and RHC. Though variable among cohorts, hemodynamic measurements are less impaired among individuals with SSc-PH-ILD as compared to SSc-PAH at baseline, and DLCO is lower than among individuals with SSc-ILD (38). In studies where patients with SSc-PH-ILD received PAH-specific therapies, there were similar improvements in hemodynamics, however, less clinical response by 6MWD and NYHA functional class.

## 4. Pathology of scleroderma-associated PAH

Pathological examination of the lung and heart in SSc-PAH yields informative and interesting observations which inform the understanding of disease pathogenesis. SSc-PAH shares many characteristic findings with IPAH such as intimal hyperplasia, medial hypertrophy, and angioproliferative lesions (39). Notably, there are fewer plexiform lesions, a hallmark of IPAH, in the lung in SSc-PAH compared to IPAH, with relatively more intimal fibrosis and venoocclusive disease (40, 41).

Pulmonary venoocclusive disease is an underrecognized cause of pulmonary vascular disease in SSc and may affect up to 50% of patients with SSc-PAH (42). Characterized by intimal proliferation of the pulmonary veins and venules, definitive diagnosis of PVOD is made by histology (43). However, given the risk of lung biopsy in patients with SSc, suspicion of this diagnosis must be high and can be suspected based on strict, non-invasive clinical criteria (44).

## 5. The importance of phenotyping pulmonary hypertension in scleroderma

Systemic sclerosis-associated pulmonary hypertension may fall under any group of the World Symposium on Pulmonary Hypertension (WSPH) classification (Table 1) (33, 45). Thus, among individuals with SSc who are found to have pulmonary hypertension by RHC, phenotyping is of utmost importance to safely direct therapy. As discussed above, remodeling of the pulmonary vasculature leads to the development of PAH (Group 1). Myocardial fibrosis and accelerated atherosclerotic disease are present at increased rates in SSc, predisposing patients to either systolic or diastolic cardiac dysfunction (Group 2, or PH diseases due to left heart disease). Although most patients with SSc develop some lung parenchymal fibrosis, the degree of involvement (as determined by pulmonary function tests combined with chest computer tomography) will determine the presence of significant ILD, with the attendant burden described above. These patients with significant ILD belong to WSPH Group 3 (PH diseases due to lung diseases and/or hypoxia). The presence of comorbid obstructive sleep apnea among patients with SSc ranges up to 50% and must be evaluated as this can contribute to changes seen in diagnostic modalities (46–49). There are also increased rates of thromboembolism which can lead to the development of chronic thromboembolic pulmonary hypertension (Group 4).

**TABLE 1:** WSPH Classification of pulmonary hypertension.

WSPH classification	Definition	Subclassification
Group I	Pulmonary arterial hypertension	1.1 Idiopathic PAH 1.2 Heritable PAH 1.3 Drug and toxin induced PAH 1.4 Associated with: connective tissue disease, HIV, portal hypertension, congenital heart disease, schistosomiasis 1.5 Long-term responders to calcium-channel blockade 1.6 Pulmonary veno-occlusive disease/pulmonary capillary hemangiomatosis 1.7 Persistent PH of the newborn syndrome
Group II	Pulmonary hypertension due to left heart disease	2.1 Heart failure with preserved left ventricular ejection fraction 2.2 Heart failure with reduced left ventricular ejection fraction 2.3 Valvular disease 2.4 Congenital or acquired cardiovascular conditions causing post-capillary PH
Group III	Pulmonary hypertension due to lung disease and/or hypoxia	3.1 Obstructive lung disease 3.2 Restrictive lung disease 3.3 Other mixed obstructive and restrictive lung diseases 3.4 Sleep disordered breathing 3.5 Alveolar hypoventilation disorders 3.6 Chronic exposure to high altitude 3.7 Congenital lung diseases
Group IV	Pulmonary hypertension due to pulmonary artery obstruction	4.1 Chronic thromboembolic pulmonary hypertension 4.2 Other pulmonary artery obstructions
Group V	Pulmonary hypertension with unclear/multifactorial mechanisms	5.1 Hematologic: myeloproliferative disorder, chronic hemolytic anemia, splenectomy 5.2 Systemic disorders (sarcoidosis, pulmonary Langerhans cell histiocytosis, lymphangioleiomyomatosis) and metabolic disorders 5.3 Other 5.4 Complex congenital heart disease

Adapted from Simonneau et al. (45).

While extensive, this form of classification, or phenotyping, carries different and important implications in terms of treatment. It is also important to note that some patients may belong to more than one WSPH group over time, with progression of their disease (e.g., fibrosis) and aging (e.g., development of heart disease such as diastolic dysfunction). From a therapeutic standpoint, vasodilator therapies, which are approved for use in Group I disease, can lead to increased fluid retention, worsening gas exchange, disease progression and higher mortality when used for Group 2/3 disease (50), particularly in PVOD where treatment with PAH-specific therapies risks causing severe pulmonary edema. An exception is inhaled treprostinil, which led to improved exercise capacity among treated patients with pulmonary hypertension due to ILD (51).

Owing to its poor prognosis, early diagnosis and treatment of SSc-PAH is critical. While not all patients with SSc develop PAH, routine screening is recommended to capture disease early in its course. The 6th World Symposium on Pulmonary Hypertension changed the hemodynamic parameters required to diagnose any form of PH, lowering cutoff mPAP from 25 to 20 mmHg, based on data from normal subjects which showed a normal mPAP was 14.0 ± 3.3 mmHg. The current definition of mild PH (mPAP between 21 and 24 mmHg) acknowledges that a mPAP > 20 mmHg is two standard deviations above this mean value in healthy individuals (45). Prospective studies have highlighted that a PVR > 2 WU was associated with significantly reduced survival, and a higher cutoff value led to inadequate diagnosis of mild PAH among patients with SSc (52). Subsequently, the 2022 ESC/ERS guidelines defined precapillary PH as a mPAP > 20 mmHg, pulmonary artery wedge pressure ≤ 15 mmHg and PVR > 2 WU (53).

Given diagnosis of PAH requires invasive testing, there has been focused interest on improved screening among patients with SSc without diagnosed PAH. A prospective study referring patients with SSC for RHC based on echocardiogram presence of tricuspid regurgitant velocity jet of > 3 m/s, or between 2.5 and 3 m/s with unexplained dyspnea found milder disease was detected at time of catheterization (54). The DETECT study developed a novel algorithm to increase the sensitivity of screening in this population using a combination of echo, PFTs, N-terminal prohormone brain natriuretic peptide (NT-proBNP) and other clinical parameters for early diagnosis (55). At our center, we perform annual screening utilizing this algorithm in patients with SSc without diagnosed PH. Guidelines recommend patients with suspected or newly diagnosed SSc-PAH be referred to a PH center of excellence for multidisciplinary management of their disease.

## 6. Unique characteristics of the right ventricle in scleroderma

In the last decade, the chasm between outcomes in IPAH and SSc-PAH has narrowed (56). However, mortality remains unacceptable high, with 5-year survival rates of 60%. Though the root cause remains unexplained, literature suggests differences in RV myocardial dysfunction may explain the discrepancy (39, 57, 58). RV stroke volume index, a surrogate of RV contractility, is lower when compared across a cohort of patients with SSc-PAH compared to IPAH, and independently predicts survival (59). Additionally, SSc-PAH patients frequently have significantly higher resting heart rate and higher NT-proBNP serum levels compared to matched patients with IPAH (60). These findings suggest a maladaptive response by the RV in response to increased pulmonary vascular load as well as differences in neurohormonal activation that is more pronounced in SSc-PAH.

Further studies of intrinsic myocardial functions support this as an explanation of worse outcomes. Endomyocardial biopsies in patients with SSc without evidence of right or left heart disease showed a significant increase in the interstitial collagen volume fraction compared with the controls (61). Further, sarcomere function as assessed by maximal calcium-activated force is depressed in early SSc-PAH, whereas it is increased in IPAH (62). In one study, patients with SSc referred for PAH evaluation were compared to healthy controls and patients with SSc in whom PAH was ruled out. Myocardial perfusion reserve indices were significantly lower in the referral group in both the right and left ventricle (63). Supporting this are findings that myocardial capillary density is reduced among patients with SSc-PAH when compared with patients with SSc without PAH and patients with HFpEF (39).

## 7. Therapy

Though data is limited in SSc-PAH, consensus guidelines for treatment of PAH recommend use of supplemental oxygen for treatment of resting or exertional hypoxia (SpO2 < 90%), use of diuretics to manage volume overload, and in certain cases use of digoxin for right heart failure which is complicated by atrial arrhythmias (21). While calcium channel blockade is commonly used for treatment of Raynaud’s phenomenon in patients with SSc, its role in treatment of PAH is recommended only for patients who demonstrate response on vasoreactivity testing during RHC, which is much less common in SSc-PAH compared to IPAH (64). Given this represents only approximately 1% of patients with SSc-PAH, calcium channel blocker use is not routinely recommended in these patients for the treatment of vasoreactive PAH (65).

Until recently, the only disease-specific treatments for WSPH Group I PAH worked via modulation of three vasodilatory pathways. Based on the understanding that a dysfunctional endothelium mediates progression of disease, medications targeted known increased levels of endothelin-1, and decreased levels of prostaglandin I-2 and nitric oxide seen in PAH.

Prostaglandin analogues potentiate adenylate cyclase-mediated conversion of ATP to cAMP which leads to vasodilation and decreased proliferation. Prostacyclin therapy with continuous intravenous epoprostenol has been shown to improve exercise capacity and hemodynamics compared with conventional therapy in patients with SSc-PAH but failed to improve survival (66). Similar findings were reported with use of continuous subcutaneous infusion of treprostinil (67, 68). Notably, inhaled treprostinil is the only FDA-approved therapy for pulmonary hypertension due to ILD (WSPH Group III).

Endothelin receptor antagonists bosentan and macitentan inhibit both the endothelin-A receptor, which effects vasoconstriction and vessel proliferation, as well as the endothelin-B receptor, which has vasodilatory actions. Ambrisentan is the only FDA-approved selective endothelin-A receptor antagonist. It has the advantage of leaving the endothelin receptor-B unopposed.

Phosphodiesterase-5 inhibitors sildenafil and tadalafil act by inhibiting conversion of cGMP to GMP, potentiating the former’s vasodilatory effects. Studies of these agents have reported improvements in 6MWD, function class, and time to clinical worsening, however, results may have been confounded by presence of other therapies and the proportion of patients with SSc-PAH in these trials was low, limiting their generalizability among this select population (69, 70). Riociguat, a soluble guanylate cyclase stimulator that acts via this pathway, showed similar improvements in exercise capacity and secondary efficacy end points and is the only agent also approved for treatment of inoperable CTEPH (71–73).

A landmark trial in PAH, AMBITION, demonstrated that combination therapy with ambrisentan and tadalafil in treatment-naïve patients was superior to monotherapy with either drug to reduce the risk of clinical failure, improve hemodynamics, and improve exercise capacity (74). A prospective, multicenter, open-label trial was performed with combination of ambrisentan and tadalafil in treatment-naïve patients with SSc-PAH and demonstrated significant improvements in functional class, hemodynamics, as well as RV structure and function by cardiac magnetic resonance imaging and two-dimensional echocardiography (75).

Recently, the phase 3 STELLAR trial performed a multicenter, double-blind, randomized and placebo-controlled comparison of subcutaneous sotatercept for the treatment of WHO functional class II or III PAH. Sotatercept, a fusion protein with the extracellular domain of human ActRIIA, functions as a ligand trap for circulating activins which are ligands to certain TGF-β superfamily receptors which have been shown to drive proliferation and inhibit apoptosis in patients in PAH. Compared to patients receiving placebo, patients who received sotatercept had a mean increase in 6MWD of 40.1 m at 24 weeks compared to −1.4 m (76, 77). However, this effect did not persist in subgroup analysis of PAH associated with connective-tissue disease, possibly due to small sample size.

Regarding SSc-specific therapies and their use in SSc-PAH, multiple studies have demonstrated treatment with B-cell depleting agent rituximab improves skin thickening in systemic sclerosis (78). A double-blinded, randomized, placebo-controlled, proof-of-concept trial in which patients with SSc-PAH received infusions of rituximab showed improvement in 6-min walk distance (6MWD) did not reach statistical significance (79). Autologous stem cell transplantation, which has been the most effective intervention to date for skin fibrosis in SSc, has not been tested for PAH (34, 80).

Lung transplantation remains the definitive treatment for PAH, however, its use in SSc-PAH is complicated by several factors. Peri-operative use of immunosuppressive medications such as mycophenolate mofetil and tacrolimus to manage extrapulmonary manifestations of SSc increases the risk of post-operative infections. Post-operative wound healing can be impaired due to the degree of skin thickening overlying the thorax. Patients with SSc have high rates of comorbid gastric and esophageal dysmotility which predisposes them to aspiration, a risk factor for chronic lung allograft dysfunction after transplantation (81). Chronic microaspiration can manifest as diffuse bronchiolitis, obliterative bronchiolitis, or exogenous lipoid pneumonia, which may complicate the pre-transplant period and obfuscate the WHO clinical grouping of PH in the absence of SSc-ILD (82). While preemptive fundoplication prior to lung transplant is controversial, this procedure is contraindicated in patients with SSc due to severe esophageal dysfunction. Despite these comorbidities, it appears that outcomes for patients with SSc-PAH who undergo lung transplantation are essentially quite comparable compared to other forms of PAH, with a 5-year survival of approximately 77% (81, 83). Patients with rapidly progressive disease, or NYHA class III or IV symptoms despite optimal medical therapy should be promptly referred for evaluation of lung transplantation (84).

## 8. Conclusion

Pulmonary arterial hypertension is a common yet devastating complication of SSc. Advances in screening have contributed to improved detection of early disease and cohort studies from the past decade show improved survival among patients with SSc-PAH compared to the decade prior. However, outcomes remain unacceptably poor, and an increased understanding of disease pathogenesis is necessary to identify additional targets for drug development. While disease-modifying therapies in PAH are emerging, the magnitude of effect on this specific population remains to be seen.

## Author contributions

TT: Visualization, Writing–original draft. PH: Conceptualization, Supervision, Writing–reviewing and editing.
